# Sodium-Glucose Cotransporter-2 Inhibitors in Valvular Heart Disease: From Mechanistic Insights to Clinical Application

**DOI:** 10.1016/j.jscai.2026.105568

**Published:** 2026-08-04

**Authors:** Haytham Allaham, C. Michael Gibson, Aloke Finn, Imad Alhaddad, Anuj Gupta

**Affiliations:** a Division of Cardiovascular Medicine, Department of Medicine, University of Maryland Medical Center, Baltimore, Maryland; b Division of Cardiovascular Medicine, Department of Medicine, Beth Israel Deaconess Medical Center, Harvard Medical School, Boston, Massachusetts; c CVPath Institute, Gaithersburg, Maryland; d Division of Cardiovascular Medicine, Jordan Hospital, Amman, Jordan

**Keywords:** cellular effects, heart failure, sodium-glucose cotransporter-2 inhibitors, systemic effects, transcatheter interventions, valvular heart disease

## Abstract

Valvular heart disease encompasses a diverse group of hemodynamic disorders that are frequently associated with heart failure, recurrent hospitalizations, and substantial morbidity despite advances in transcatheter and surgical therapies. Sodium-glucose cotransporter-2 (SGLT2) inhibitors have emerged as a cornerstone of cardiovascular and renal disease management, demonstrating consistent benefits across a broad range of patient populations. Beyond their established effects in heart failure, diabetes mellitus, and chronic kidney disease, accumulating evidence suggests that SGLT2 inhibitors may favorably influence key biological pathways involved in valvular heart disease, including inflammation, oxidative stress, fibrosis, endothelial dysfunction, and adverse cardiac remodeling. Preclinical and translational studies have provided mechanistic support for a potential role of SGLT2 inhibition in modifying valvular and myocardial disease processes. Emerging clinical evidence suggests possible benefits across several forms of valvular heart disease, including degenerative aortic stenosis, functional mitral regurgitation, tricuspid regurgitation, rheumatic mitral stenosis, and bioprosthetic valve degeneration. In addition, growing interest has focused on the integration of SGLT2 inhibitors into contemporary structural heart practice, particularly among patients undergoing transcatheter aortic valve replacement, transcatheter edge-to-edge repair, and other structural interventions. This narrative review summarizes the mechanistic rationale, current clinical evidence, and practical considerations surrounding the use of SGLT2 inhibitors in valvular heart disease and structural heart interventions. We discuss the strengths and limitations of the existing literature, identify important gaps in knowledge, and highlight future directions for research aimed at defining the role of SGLT2 inhibitors as an adjunctive therapy in structural heart disease.

## Introduction

Sodium-glucose cotransporter-2 (SGLT2) inhibitors have become foundational therapy for heart failure (HF), with randomized clinical trials demonstrating reductions in HF hospitalization and cardiovascular mortality across the spectrum of left ventricular ejection fraction and irrespective of diabetes status.^1^, ^2^, ^3^, ^4^, ^5^, ^6^ However, patients with valvular heart disease (VHD), particularly those with significant valvular pathology or undergoing valve intervention, were largely excluded from these pivotal trials; when patients with mild or moderate VHD were permitted, their representation and outcomes were inconsistently reported.^1^, ^2^, ^3^, ^4^, ^5^, ^6^ This limits the generalizability of trial findings to VHD populations, who represent a distinct clinical substrate characterized by chronic pressure or volume overload, progressive valvular disease, and secondary myocardial remodeling. Despite extensive literature on SGLT2 inhibitors in HF, their role in valvular VHD remains incompletely defined. This review summarizes the current evidence supporting SGLT2 inhibitor therapy in VHD, including systemic and cellular mechanisms, potential applications across the disease spectrum, adjunctive use around valve interventions, and remaining knowledge gaps and future research priorities (Central Illustration).

**Central Illustration:**
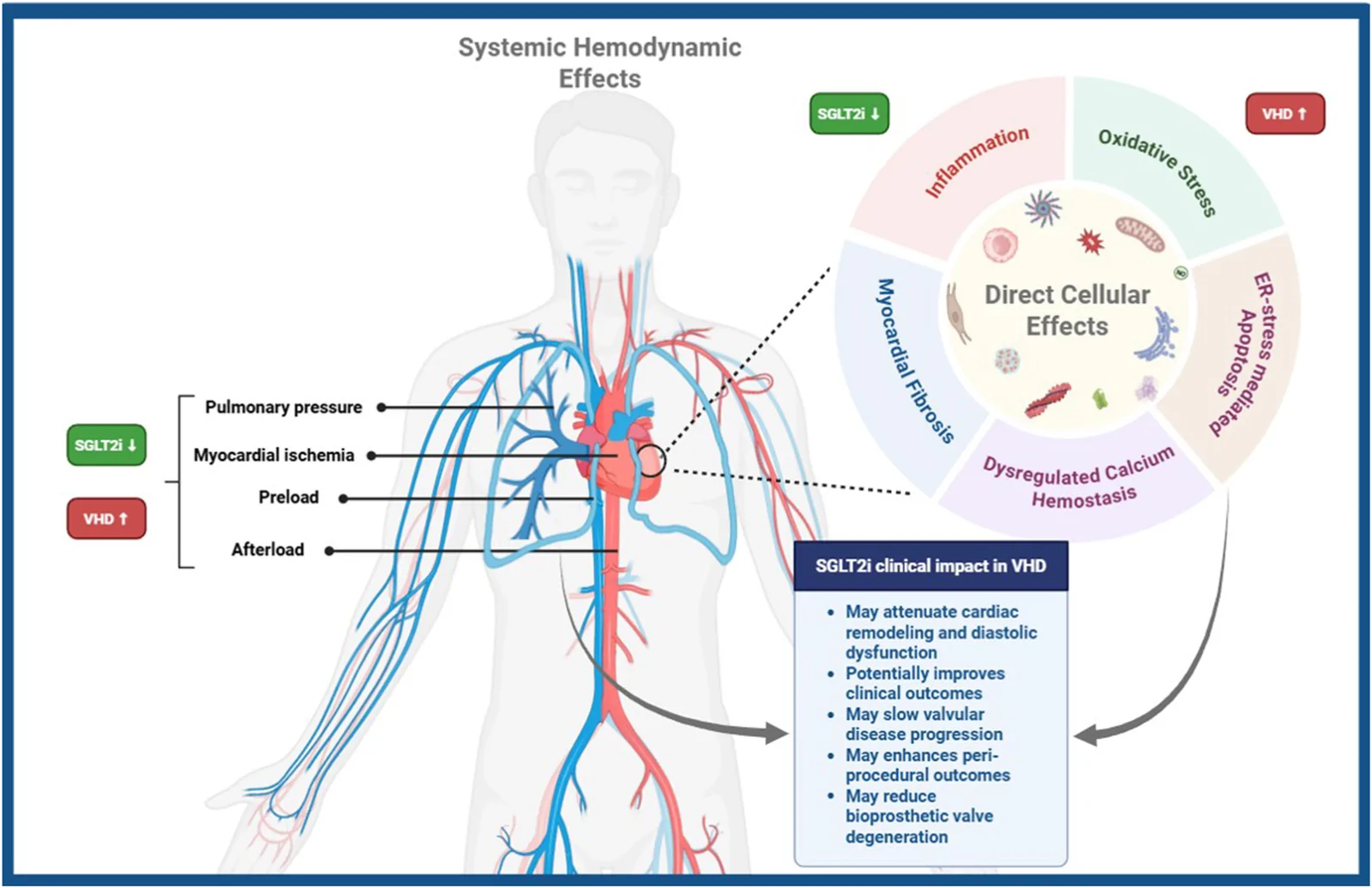
**Conceptual framework illustrating how SGLT2 inhibition may influence VHD progression through complementary systemic hemodynamic and direct cellular mechanisms.** These pathways collectively may mitigate myocardial remodeling, modulate valvular pathology, and may contribute to improved clinical outcomes across the spectrum of VHD. ER, endoplasmic reticulum; SGLT2i, sodium-glucose cotransporter-2 inhibitors; VHD, valvular heart disease.

## Methods

This narrative review was informed by a literature search designed to identify mechanistic, translational, and clinical studies evaluating the role of SGLT2 inhibitors in VHD.

Electronic databases, including PubMed/MEDLINE, PubMed Central, Embase (Elsevier), Web of Science (Clarivate Analytics), Scopus (Elsevier), and the Cochrane Central Register of Controlled Trials (CENTRAL), were searched. Additional relevant publications were identified through manual review of reference lists of key articles and targeted supplementary searches using Google Scholar.

Search terms included “sodium-glucose cotransporter-2 inhibitors,” “SGLT2 inhibitors,” and “SGLT2i” combined with terms related to structural heart disease and valvular pathology, including “structural heart disease,” “valvular heart disease,” “aortic stenosis,” “mitral regurgitation,” “mitral stenosis,” “tricuspid regurgitation,” “transcatheter aortic valve replacement,” “aortic valve replacement,” “transcatheter mitral edge-to-edge repair,” “transcatheter tricuspid edge-to-edge repair,” “cardiac surgery,” and “structural heart interventions.” Additional terms related to systemic hemodynamic effects, myocardial remodeling, and cellular mechanisms were included to capture relevant experimental and translational studies.

Two investigators (H.A. and A.G.) independently screened titles and abstracts for relevance. Studies considered eligible included experimental investigations, observational cohort studies, randomized clinical trials, and relevant translational research evaluating mechanistic or clinical effects of SGLT2 inhibition in VHD or structural heart interventions. Full-text review was performed for articles deemed potentially relevant.

No restrictions were applied regarding publication date or geographic region. Studies published through February 2026 were considered eligible, and only articles published in the English language were included. Because of heterogeneity in study design, patient populations, and reported outcomes, findings were synthesized qualitatively using a narrative approach with emphasis on mechanistic insights, clinical outcomes, and implications for structural heart interventions.

This review was based exclusively on previously published literature and did not involve new data collection from human participants or animals.

## Systemic hemodynamic and metabolic effects of SGLT2 inhibitors in VHD

**Figure 1:**
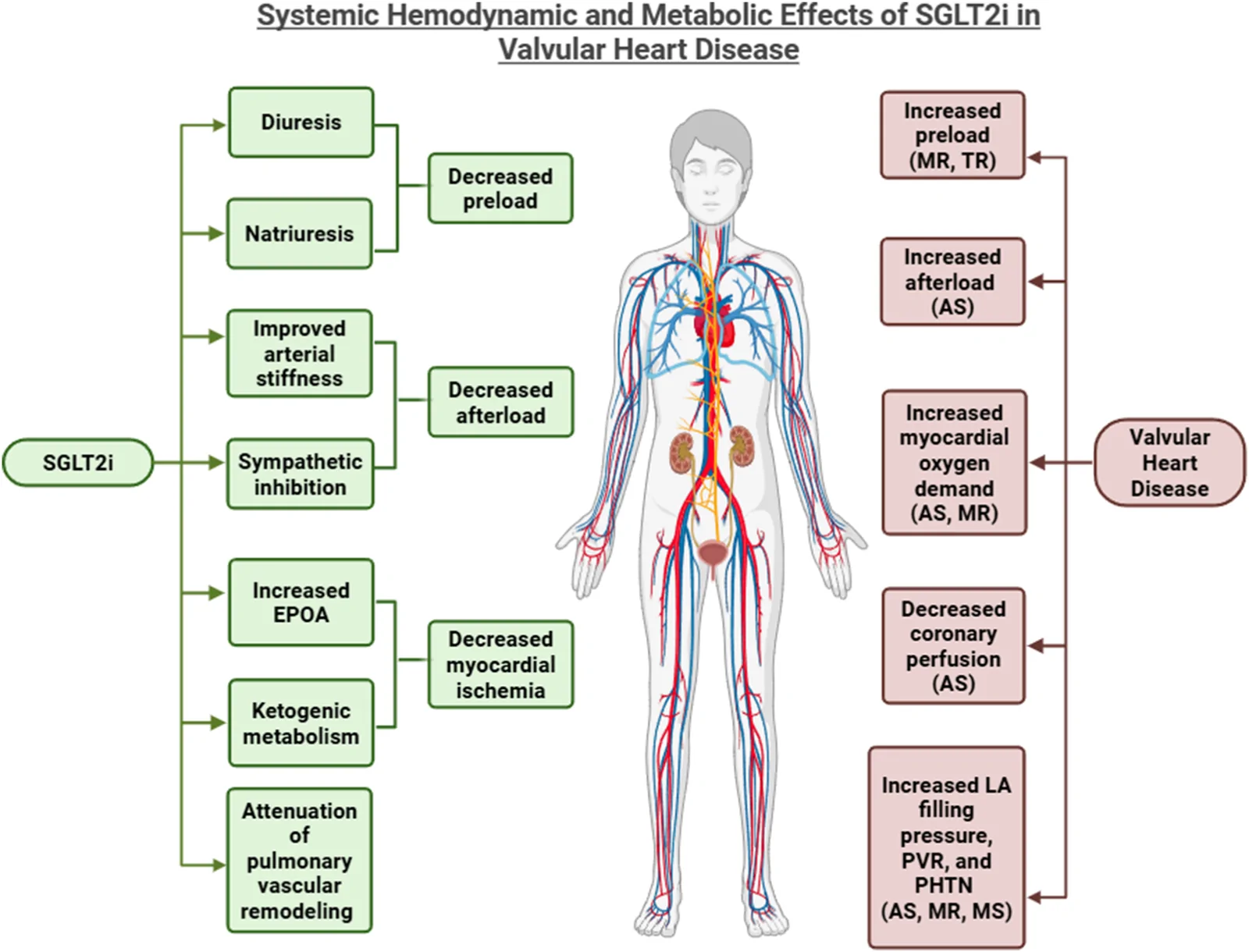
**SGLT2 inhibitors exert multimodal cardioprotective effects throughnatriuresis-mediatedpreload reduction, vascular and autonomic modulation leading to afterload improvement, and favorable pulmonary vascular effects.** In parallel, metabolic shifts toward ketone utilization and stimulation of erythropoiesis enhance myocardial energetic efficiency and oxygen delivery. Collectively, these mechanisms may counterbalance key pathophysiologic pathways driving valvular heart disease progression. AS, aortic stenosis; LA, left atrial; MR, mitral regurgitation; MS, mitral stenosis; PHTN, pulmonary hypertension; PVR, pulmonary vascular resistance; SGLT2i, sodium-glucose cotransporter-2 inhibitors; TR, tricuspid regurgitation.

Cardiac anatomy and hemodynamics exist in a tightly integrated and dynamically balanced relationship under physiological conditions. VHD disrupts this equilibrium through progressive anatomic alterations that drive maladaptive hemodynamic changes and myocardial remodeling (Figure 1). Over the past 2 decades, therapeutic strategies in VHD have largely focused on correcting the underlying structural abnormality through surgical or transcatheter interventions, both of which have demonstrated meaningful improvements in clinical outcomes and symptom burden.^7^, ^8^, ^9^ These interventions are also associated with favorable hemodynamic changes and reverse cardiac remodeling following treatment.^10^, ^11^, ^12^ More recently, SGLT2 inhibitors have emerged as a potential therapeutic strategy that may favorably influence the systemic hemodynamic processes contributing to VHD progression.^13^

SGLT2 inhibitors exert a diuretic effect primarily through inhibition of sodium reabsorption in the proximal convoluted tubule, resulting in natriuresis and modest contraction of effective circulating volume.^14^ These preload-reducing effects may contribute to lower ventricular filling pressures and are particularly advantageous in conditions characterized by chronic volume overload, such as mitral regurgitation (MR) and tricuspid regurgitation (TR), where sustained elevations in preload promote ventricular and atrial dilation as well as progressive diastolic dysfunction.^15^ Additionally, increased glycosuria induces osmotic diuresis, further augmenting volume reduction. Notably, emerging data suggest that SGLT2i-mediated volume loss preferentially affects the interstitial compartment, with comparatively smaller reductions in intravascular volume.^16^ This profile confers advantages over loop diuretics, which are often associated with reflex neurohormonal activation and tachycardia. Such hemodynamic characteristics may be particularly beneficial in VHD populations, where myocardial oxygen supply–demand imbalance is frequently present.

Beyond preload modulation, accumulating evidence indicates that SGLT2 inhibitors exert favorable afterload-reducing effects, partly mediated through attenuation of sympathetic nervous system activity.^17^^,^^18^ Reduction in afterload is particularly relevant in VHD states characterized by chronically elevated left ventricular pressure load, such as aortic stenosis (AS), where sustained afterload contributes to compensatory left ventricular hypertrophy, progressive diastolic dysfunction, and eventual impairment in cardiac output. In addition, SGLT2 inhibitors have been associated with improvements in arterial stiffness through multiple mechanisms, leading to reductions in central systolic blood pressure and pulse pressure.^19^^,^^20^ These vascular effects offer additional hemodynamic benefit across VHD phenotypes, including both AS and MR.

Pulmonary vascular dysfunction represents a critical pathophysiologic component across multiple VHD states. Emerging evidence suggests that SGLT2 inhibitors may favorably influence pulmonary hemodynamics through reductions in pulmonary artery pressures, including mean pulmonary artery pressure and pulmonary artery systolic pressure, as well as attenuation of pulmonary vascular remodeling.^21^ These effects hold particular clinical relevance in conditions such as MR, mitral stenosis (MS), and TR, where the development and severity of pulmonary hypertension are closely linked to symptom burden, prognosis, and candidacy for interventional therapies.

From a metabolic standpoint, SGLT2 inhibitors promote a metabolic shift toward mild ketogenic metabolism, characterized by increased myocardial utilization of ketone bodies as an energy substrate.^22^ Ketone oxidation is considered more oxygen-efficient compared with fatty acid and glucose metabolism, potentially enhancing myocardial energetic efficiency. This metabolic adaptation represents an additional mechanism through which SGLT2 inhibitors may confer cardioprotective effects in VHD.

Finally, clinical evidence suggests that SGLT2 inhibitors promote erythropoiesis through enhanced renal erythropoietin secretion.^23^ Subsequent increases in erythrogenesis and improved iron utilization confer favorable myocardial effects by augmenting tissue oxygen delivery and potentially mitigating the myocardial oxygen supply–demand mismatch observed across the spectrum of VHD.

## Direct cellular effects of SGLT2 inhibitors in VHD

**Figure 2:**
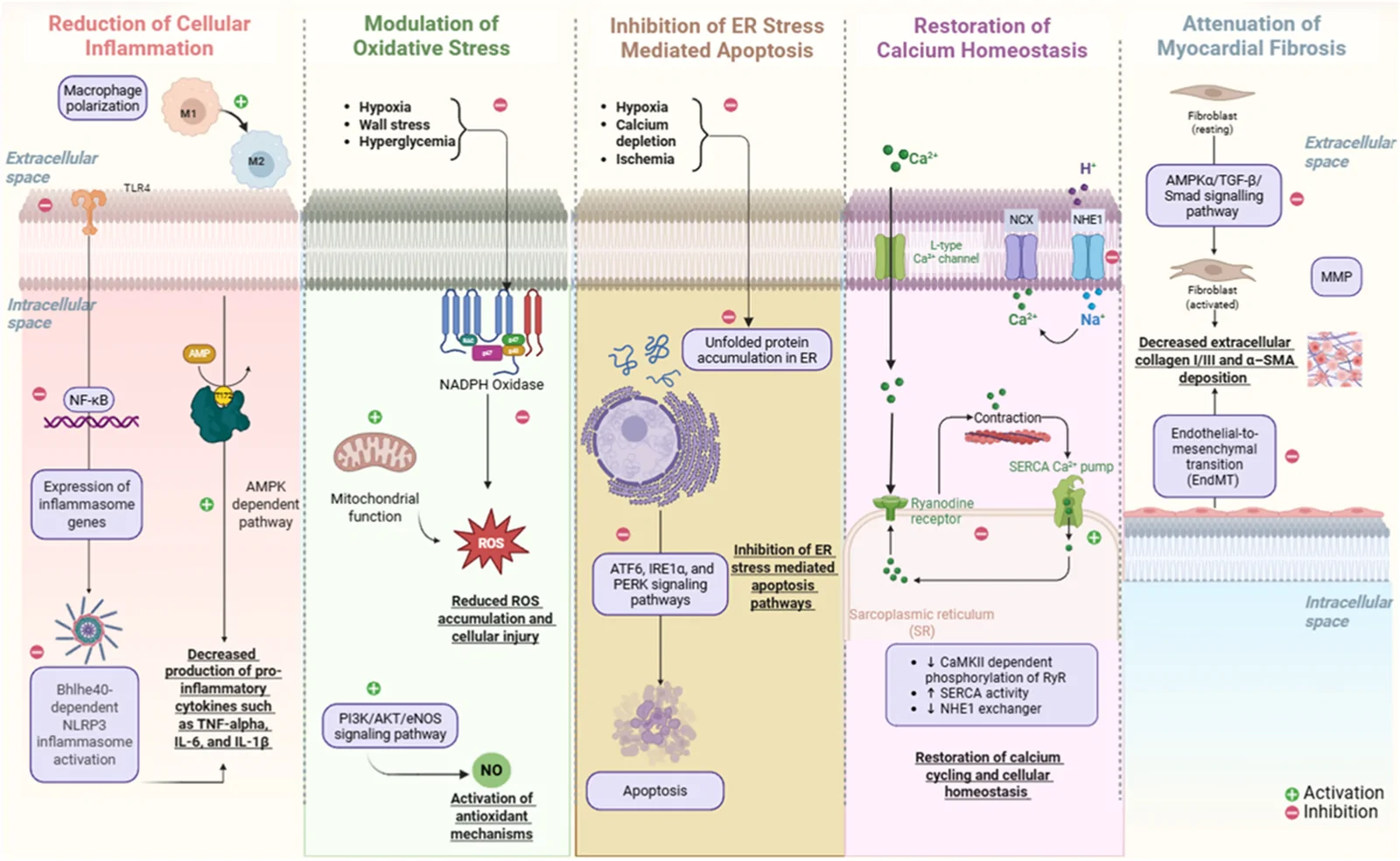
**Overview of proposed cellular mechanisms through which SGLT2i may exert cardioprotective effects in valvular heart disease.** SGLT2i influence key intracellular and extracellular pathways leading to reduction of inflammation and oxidative stress, inhibition of endoplasmic reticulum–mediated apoptosis, restoration of intracellular calcium homeostasis, and reduction of myocardial fibrosis, collectively counteracting the maladaptive cellular effects of chronic pressure and volume overload. AMPK, AMP-activated protein kinase; ATF6, activation transcription factor 6; eNOS, endothelial NO synthase; ER, endoplasmic reticulum; IL-1B, interleukin-1B; IL-6, interleukin-6; IRE1α, inositol-requiring enzyme 1 alpha; M1, M1 macrophage; M2, M2 macrophage; MMP, matrix metalloproteinase; NCX, sodium-calcium exchanger; NF-κB, nuclear factor-kappa B; NHE1, sodium-hydrogen exchanger 1; NO, nitric oxide; PERK, protein kinase R-like ER kinase; PI3K, phosphatidylinositol 3-kinase; ROS, reactive oxygen species; TGF-β, transforming growth factor beta; TNF-α, tumor necrosis factor-alpha.

Beyond hemodynamic alterations, VHD is associated with myocardial dysfunction at the cellular level. Preclinical animal models and human cardiomyocyte studies have identified multiple intracellular pathways through which SGLT2 inhibitors exert cardioprotective effects, potentially counteracting the maladaptive cellular processes observed in VHD (Figure 2).

### Reduction of cellular inflammation

Inflammatory and immune-mediated pathways play an important role in the pathogenesis of VHD, contributing to progressive valvular remodeling and secondary myocardial dysfunction.^24^, ^25^, ^26^ Preclinical studies indicate that SGLT2 inhibitors may exert anti-inflammatory effects through several complementary mechanisms, including activation of adenosine monophosphate-activated protein kinase (AMPK), a key regulator of cellular energy homeostasis and inflammatory signaling.^27^, ^28^, ^29^ Studies have shown that SGLT2 inhibition may modulate the nuclear factor-kappa B (NF-κB) signaling pathway, downregulate Toll-like receptor 4 expression, and suppress basic helix-loop-helix family member E40 (Bhlhe40)-dependent NOD-like receptor protein 3 (NLRP3) inflammasome activation, leading to reduced production of proinflammatory cytokines such as tumor necrosis factor-α (TNF-α), interleukin (IL)-6, and IL-1β.^30^, ^31^, ^32^ In addition, SGLT2 inhibitors have been shown to potentially promote macrophage polarization from the proinflammatory M1 phenotype toward the anti-inflammatory M2 phenotype, further attenuating inflammatory signaling.^33^^,^^34^ Experimental studies have demonstrated that SGLT2 inhibition may reduce NLRP3 inflammasome activity and decrease expression of osteogenic calcification markers within stenotic valves, suggesting a potential role in mitigating inflammation-driven valvular calcification and slowing disease progression in VHD.^35^

### Modulation of oxidative stress–mediated cellular injury

Chronic pressure and volume overload in VHD promotes oxidative stress through excessive generation of reactive oxygen species (ROS) within cardiomyocytes, driven by hypoxia, increased metabolic demand, and mechanical strain.^36^, ^37^, ^38^, ^39^, ^40^, ^41^ This process is further amplified by mitochondrial dysfunction and impaired intrinsic antioxidant defenses, resulting in ROS-mediated cellular injury and myocardial fibrosis.^42^^,^^43^ In addition to myocardial remodeling, activation of nicotinamide adenine dinucleotide phosphate (NADPH) oxidase in experimental models promotes differentiation of aortic valvular interstitial cells toward an osteogenic phenotype, contributing to valvular calcification through ROS-dependent pathways.^44^ Preclinical studies demonstrate that SGLT2 inhibitors have been associated with attenuation of oxidative stress by inhibiting NADPH oxidase activity and preserving mitochondrial integrity, thereby possibly reducing intracellular ROS accumulation.^45^, ^46^, ^47^ In parallel, animal studies suggested activation of the phosphoinositide 3-kinase (PI3K)/protein kinase B (AKT)/endothelial nitric oxide synthase (eNOS) signaling pathway by SGLT inhibitors, which increases nitric oxide bioavailability, further limiting oxidative injury and adverse myocardial remodeling in pressure and volume overload states.^48^, ^49^, ^50^

### Inhibition of Endoplasmic reticulum stress–mediated apoptosis

Endoplasmic reticulum (ER) stress has emerged as an important contributor to myocardial dysfunction in VHD, particularly in the setting of chronic pressure and volume overload associated with elevated filling pressures.^51^^,^^52^ Under conditions of oxidative stress, hypoxia, or calcium depletion, accumulation of misfolded proteins activates the unfolded protein response, a compensatory pathway that attempts to restore cellular homeostasis.^53^ When these adaptive mechanisms are overwhelmed, persistent ER stress triggers apoptosis through activation of multiple signaling cascades, contributing to diastolic and systolic dysfunction, dysregulated ion homeostasis, and increased arrhythmia susceptibility.^54^^,^^55^ Preclinical studies suggest that SGLT2 inhibitors mitigate ER stress–mediated injury. In experimental models of MR-induced myocardial dysfunction, SGLT2 inhibition reduced ER stress- and apoptosis-related protein expression, attenuated myocardial fibrosis and arrhythmogenesis, and partially restored ventricular function, supporting ER stress modulation as an additional mechanism by which SGLT2 inhibitors may limit maladaptive remodeling in VHD.^56^, ^57^, ^58^

### Restoration of intracellular calcium homeostasis

Intracellular calcium homeostasis is tightly regulated under physiological conditions but becomes disrupted in VHD, where chronic pressure and volume overload promote maladaptive remodeling and impaired excitation-contraction coupling.^59^^,^^60^ Experimental models of pressure overload demonstrate significant alterations in cardiomyocyte calcium handling, including reduced peak systolic Ca^2+^ transients, abnormal calcium cycling kinetics, and prolongation of the action potential due to changes in ion channels, pumps, and exchangers.^61^ Preclinical studies demonstrated that SGLT2 inhibitors favorably modulate key calcium handling and metabolic regulatory proteins, including sarcoplasmic reticulum Ca^2+^-ATPase (SERCA2a), Ca^2+^/calmodulin-dependent protein kinase II–mediated regulation of the ryanodine receptor, and the sodium-hydrogen exchanger, suggesting restoration of intracellular calcium cycling and cellular homeostasis.^50^^,^^62^, ^63^, ^64^, ^65^ These effects have been associated with improved myocardial contractility, reductions in cardiac chamber remodeling, and decreased arrhythmia susceptibility in experimental models, mechanisms that may be particularly relevant in advanced VHD.^66^, ^67^, ^68^, ^69^

### Attenuation of myocardial fibrosis

Transforming growth factor-β1 (TGF-β1)–mediated fibroblast activation represents a central profibrotic pathway driving myocardial and valvular remodeling across the spectrum of pressure and volume overload states commonly present in VHD.^70^, ^71^, ^72^, ^73^, ^74^, ^75^ SGLT2 inhibitors appear to attenuate myocardial fibrosis through modulation of multiple interconnected intracellular signaling pathways. Beyond these intracellular effects, SGLT2 inhibitors also influence extracellular matrix remodeling through several complementary mechanisms. In vitro models demonstrate that SGLT2 inhibitors attenuate TGF-β1–induced fibroblast activation, perivascular endothelial-to-mesenchymal transition, and extracellular matrix remodeling through modulation of matrix metalloproteinase activity.^76^, ^77^, ^78^, ^79^, ^80^ This results in downregulation of key profibrotic extracellular mediators, including type I and III collagen, α-smooth muscle actin, and connective tissue growth factor (TGF). These antifibrotic effects may contribute to attenuation of maladaptive myocardial remodeling, interstitial fibrosis, and diastolic dysfunction, processes that are closely linked to adverse clinical outcomes in VHD.^81^, ^82^, ^83^, ^84^

Taken together, beyond their systemic hemodynamic effects, SGLT2 inhibitors can potentially exert direct cardioprotective actions at the cellular level in VHD. Although experimental studies provide a mechanistic rationale for potential direct cellular effects of SGLT2 inhibitors, most of these data are derived from preclinical animal models and in vitro cardiomyocyte or valvular interstitial cell studies. Therefore, findings related to inflammation, oxidative stress, NLRP3 inflammasome signaling, fibrosis, calcification, and myocardial remodeling should be interpreted as hypothesis generating rather than definitive evidence of disease modification in human VHD. Importantly, whether these cellular effects translate into clinically meaningful slowing of valvular calcification, attenuation of adverse remodeling, or improved valve-related outcomes in patients remains unproven and requires validation in dedicated translational studies and adequately powered randomized clinical trials.

## Impact of SGLT2 inhibitors on disease progression and clinical outcomes in VHD

The aforementioned cellular and hemodynamic mechanisms may partially account for the emerging clinical signals observed with SGLT2 inhibitor therapy in VHD. Early clinical evidence—primarily derived from observational studies—suggests that SGLT2 inhibitors may influence disease progression and remodeling across several forms of VHD (Table 1).

**Table 1:** Clinical studies evaluating sodium-glucose cotransporter-2 inhibitors across the spectrum of valvular heart disease and structural heart interventions.

Study/author	Year	Study design	Sample size	SGLT2 inhibitor	VHD/intervention	Key clinical/echocardiographic outcomes	Follow-up
SGLT2 inhibitors in VHD							
Shah et al^85^	2025	Observational cohort study	11,698 (458 SGLTi vs no SGLTi 11,240)	Class effect	Degenerative AS	Slower progression of AS severity	36 mo
Morel et al^86^	2026	Propensity-matched retrospective cohort study	21,824 (10,912 SGLTi vs 10,912 no SGLTi)	Class effect	Degenerative AS	Reduced all-cause mortality and lower rates of TAVR/SAVR	14 mo
EFFORT^87^	2024	Multicenter double-blind randomized controlled trial	128 (63 ertugliflozin vs 65 placebo)	Ertugliflozin	Functional MR	Reduced MR severity with improved LA remodeling and LV strain	12 mo
DEFORM^88^	2025	Prospective randomized controlled trial	104 (52 dapagliflozin vs 52 placebo)	Dapagliflozin	Functional MR	Reduced MR severity and myocardial remodeling; nonsignificant trend toward lower HF hospitalizations/CV death	3 mo
Asrial et al^89^	2023	Open-label randomized controlled trial	33 (17 dapagliflozin vs 16 placebo)	Dapagliflozin	Rheumatic MS	Improved LA function, reduced mitral gradient, and greater NT-proBNP reduction	1 mo
Loutati et al^90^	2025	Observational cohort study	28,940	Class effect	TR in HF	Reduced HF hospitalization and all-cause mortality across TR severity strata	42 mo
SGLT2 inhibitors in structural heart interventions							
DAPA-TAVI^95^	2025	Multicenter open-label randomized controlled trial	1222 (607 dapagliflozin vs 615 placebo)	Dapagliflozin	TAVR	Reduced composite of all-cause mortality or worsening heart failure	12 mo
Yasmin et al^93^	2026	Propensity-matched registry-based study	4078 (2039 SGLTi vs 2039 no SGLTi)	Class effect	TAVR	Reduced HF hospitalization and mortality following TAVR	12 mo
Abbas et al^96^	2025	Propensity-matched retrospective cohort study	83 (SGLTi vs 332 no SGLTI)	Class effect	TAVR/SAVR	Reduced incidence of bioprosthetic structural valve degeneration	57 mo
Thakkar et al^97^	2024	Propensity-matched registry-based study	2578 (1289 SGLTi vs 1289 no SGLTi)	Class effect	M-TEER	Reduced composite of all-cause mortality or heart failure hospitalization	12 mo

CV, cardiovascular; HF, heart failure; LV, left ventricular; MR, mitral regurgitation; MS, mitral stenosis; M-TEER, mitral transcatheter edge-to-edge repair; SGLT2i, sodium-glucose cotransporter-2 inhibitors; TAVR, transcatheter aortic valve replacement; TR, tricuspid regurgitation.

Among patients with nonsevere AS, retrospective cohort analyses provide preliminary evidence supporting a potential association between SGLT2 inhibitor therapy and slower disease progression. In an observational study by Shah et al,^85^ patients with nonsevere AS receiving SGLT2 inhibitors were less likely to progress to severe disease on transthoracic echocardiography over 12 months of follow-up. Notably, among nearly 12,000 patients included in the study, only 458 received SGLT2 inhibitor therapy, highlighting the current underutilization of these agents in routine practice. Consistent with these findings, Morel et al^86^ reported that SGLT2 inhibitor use was associated with slower progression of degenerative AS and a lower incidence of subsequent aortic valve intervention, either surgical or transcatheter. In addition, SGLT2 inhibitor therapy was associated with reductions in all-cause mortality and progression to end-stage renal disease. However, both studies were retrospective and remain vulnerable to treatment-selection bias, residual confounding, and immortal time bias. In addition, the Morel et al^86^ analysis relied largely on clinical endpoints rather than systematic longitudinal echocardiographic assessment, limiting conclusions regarding true hemodynamic modification of AS progression. Although these findings suggest a potential disease-modifying signal, they should be interpreted cautiously pending confirmation in adequately powered randomized trials with standardized imaging follow-up.

Higher-level evidence supporting the role of SGLT2 inhibition in VHD has begun to emerge in patients with functional MR. The randomized, double-blind, multicenter EFFORT (Ertugliflozin for Functional Mitral Regurgitation Associated With Heart Failure) trial evaluated ertugliflozin in patients with HF and functional MR and demonstrated significant improvements in left ventricular global longitudinal strain and favorable left atrial remodeling.^87^ These structural changes were accompanied by reductions in MR severity, reflected by decreases in effective regurgitant orifice area and regurgitant volume at 12 months. Collectively, these findings suggest that SGLT2 inhibition may favorably influence ventricular and atrial remodeling processes that contribute to secondary MR. However, the trial was modest in size and primarily assessed echocardiographic remodeling endpoints rather than hard clinical outcomes. Therefore, while these findings support SGLT2 inhibitors as a potential component of medical optimization in functional MR, they should be interpreted as hypothesis generating pending larger trials with longer follow-up.

Additional support comes from the DEFORM (Dapagliflozin Effect on Functional Mitral Regurgitation and Myocardial Remodelling) trial, which evaluated dapagliflozin in patients with functional moderate-to-severe MR receiving optimal guideline-directed medical therapy.^88^ In this randomized study, SGLT2 inhibitor therapy was associated with reductions in MR severity and favorable changes in left ventricular remodeling parameters. Although a numerical reduction in HF hospitalizations and cardiovascular mortality was observed in the SGLT2 inhibitor group, these differences did not reach statistical significance, likely reflecting the relatively small sample size. Importantly, the trial was also limited by the short 3-month follow-up and reliance primarily on echocardiographic remodeling endpoints. These limitations restrict conclusions regarding the durability of MR improvement and whether these imaging changes translate into meaningful clinical benefit. Nonetheless, the findings provide additional clinical signals suggesting that modulation of ventricular remodeling with SGLT2 inhibition may influence the ventricular substrate that drives functional MR.

Evidence in rheumatic MS remains limited but similarly suggests potential hemodynamic benefits. In an open-label randomized study, Asrial et al^89^ demonstrated that dapagliflozin therapy was associated with increased net atrioventricular compliance, reductions in mean transmitral gradient, and significant declines in N-terminal pro-B-type natriuretic peptide (NT-proBNP) levels at 4 weeks of follow-up. Biomarkers of fibrosis, including procollagen type I C-terminal propeptide, the matrix metalloproteinase-1 (MMP-1)/tissue inhibitor of metalloproteinases 1 (TIMP-1) ratio, and TGF-β1-did not significantly change, suggesting that the observed hemodynamic improvements may have been driven primarily by preload and ventricular loading effects rather than direct antifibrotic mechanisms. To date, there are no dedicated studies evaluating the safety or clinical efficacy of SGLT2 inhibitors in patients with nonrheumatic MS, including calcific MS, and their potential role in this population remains undefined.

Clinical evidence evaluating SGLT2 inhibitors in TR remains sparse. In a retrospective cohort analysis of patients with HF and concomitant TR, Loutati et al^90^ reported that SGLT2 inhibitor therapy was associated with lower risks of hospitalization and mortality across the spectrum of TR severity. Prospective studies, including the ongoing EVENT trial (Enavogliflozin Outcome Trial in Functional TR; NCT06027307), are expected to further clarify the potential role of SGLT2 inhibition in this population.

Taken together, emerging clinical data, supported by mechanistic and translational evidence, suggest that SGLT2 inhibition may exert favorable effects across multiple components of VHD, including ventricular remodeling, hemodynamic burden, and potentially valvular disease progression. However, much of the current evidence remains *exploratory* and is largely derived from observational or early-phase studies. Larger randomized trials are therefore required to determine whether these signals translate into durable improvements in clinical outcomes and meaningful modification of valvular disease progression.

## SGLT2 inhibitors as adjunct therapy to transcatheter structural heart interventions

Transcatheter and surgical valve interventions have advanced substantially over the past 2 decades across the spectrum of VHD. Despite favorable procedural outcomes, recurrent HF hospitalizations remain a significant clinical and economic burden.^91^^,^^92^ The concept of adjunctive medical therapy following correction of the mechanical lesion has been previously explored in this high-risk population. Recent studies suggest a potential role for SGLT2 inhibitors in improving outcomes among these patients (Table 1).

The beneficial effects of SGLT2 inhibitors in structural heart interventions are most robustly supported in the transcatheter aortic valve replacement (TAVR) population. Several observational studies highlight the potential role for these agents in reducing recurrent HF hospitalizations following TAVR.^93^^,^^94^ These observations were further supported by the DAPA-TAVI (Dapagliflozin in Patients Undergoing Transcatheter Aortic-Valve Implantation) trial, which randomly assigned nearly 1200 patients undergoing TAVR to dapagliflozin or placebo.^95^ In addition to severe AS, eligible participants were required to have at least 1 high-risk feature, including renal insufficiency, diabetes mellitus, or left ventricular systolic dysfunction. Treatment with dapagliflozin was associated with a significant reduction in the primary composite endpoint of all-cause mortality and recurrent HF events within 1 year following the procedure. This benefit was largely driven by a decrease in HF hospitalizations, while no statistically significant difference in all-cause mortality was observed between groups.

While DAPA-TAVI represents the highest-quality evidence currently available in this field, several limitations warrant consideration. The trial employed an open-label design, which may have influenced clinical decision-making and reporting of softer endpoints, such as HF hospitalizations. In addition, the study population was restricted to patients with severe AS and additional high-risk comorbidities, potentially limiting the generalizability of the findings to lower-risk TAVR populations and other forms of structural heart disease. Furthermore, the relatively short 1-year follow-up precludes assessment of long-term effects on ventricular remodeling, valve durability, and survival.

Recent observational data suggest a potential protective role for SGLT2 inhibitors in patients receiving both transcatheter and surgical bioprosthetic aortic valves.^96^ In a propensity-matched retrospective analysis of nearly 650 patients, Abbas et al^96^ demonstrated that the 10-year cumulative incidence of structural valve degeneration was observed to be lower among patients treated with SGLT2 inhibitors compared with those not receiving these agents. However, the small size of the exposed cohort, nonrandomized treatment allocation, and potential for residual confounding and immortal time bias limit causal inference. While these findings remain hypothesis generating, they raise the intriguing possibility that SGLT2 inhibitors may influence not only myocardial remodeling but also the long-term durability of bioprosthetic valves. Prospective studies are warranted to confirm these observations and to better define the role of SGLT2 inhibition in patients undergoing surgical valve replacement.

Evidence specifically evaluating SGLT2 inhibitors in the context of both mitral and tricuspid transcatheter edge-to-edge repair (M-TEER and T-TEER) remains limited, although guideline-directed medical therapy is well established in patients with MR and TR. Notably, SGLT2 inhibitors were not incorporated into landmark M-TEER trials, including the COAPT (Cardiovascular Outcomes Assessment of the MitraClip Percutaneous Therapy for Heart Failure Patients With Functional Mitral Regurgitation) trial.^9^ Available data are largely derived from small observational studies suggesting potential associations between SGLT2 inhibitor use and reductions in HF hospitalization and selected adverse clinical events following M-TEER; however, these findings are inherently hypothesis generating.^97^^,^^98^ Evidence in the T-TEER population is even more sparse, with only preliminary retrospective reports available.^99^ Collectively, these gaps underscore a substantial unmet need for adequately powered, prospective randomized clinical trials to define the efficacy and safety of SGLT2 inhibitor therapy in patients undergoing both M-TEER and T-TEER.

## Perioperative management of SGLT2 inhibitors in surgical valve interventions

Finally, the role of SGLT2 inhibitors in cardiac surgery has begun to emerge, with early observational data suggesting potential outcome improvement.^100^^,^^101^ However, these studies have not consistently stratified results according to valve type or timing of therapy initiation relative to surgery, limiting interpretation and generalizability. A perioperative concern has been the risk of acute kidney injury with SGLT2 inhibitor exposure; nevertheless, recent studies indicate that SGLT2 inhibitor use may be associated with nephroprotective effects in patients undergoing cardiac surgery with extracorporeal circulation.^102^^,^^103^

Additional reports suggest that perioperative discontinuation of SGLT2 inhibitors in patients with HF may be associated with higher rates of postoperative HF hospitalization.^104^^,^^105^ Collectively, these findings suggest potentially a reassuring safety profile and a possible clinical benefit of perioperative SGLT2 inhibitor therapy, although the observational nature of available data and heterogeneity in perioperative management strategies underscore the need for prospective randomized studies to define optimal patient selection and timing of initiation.

**Figure 3:**
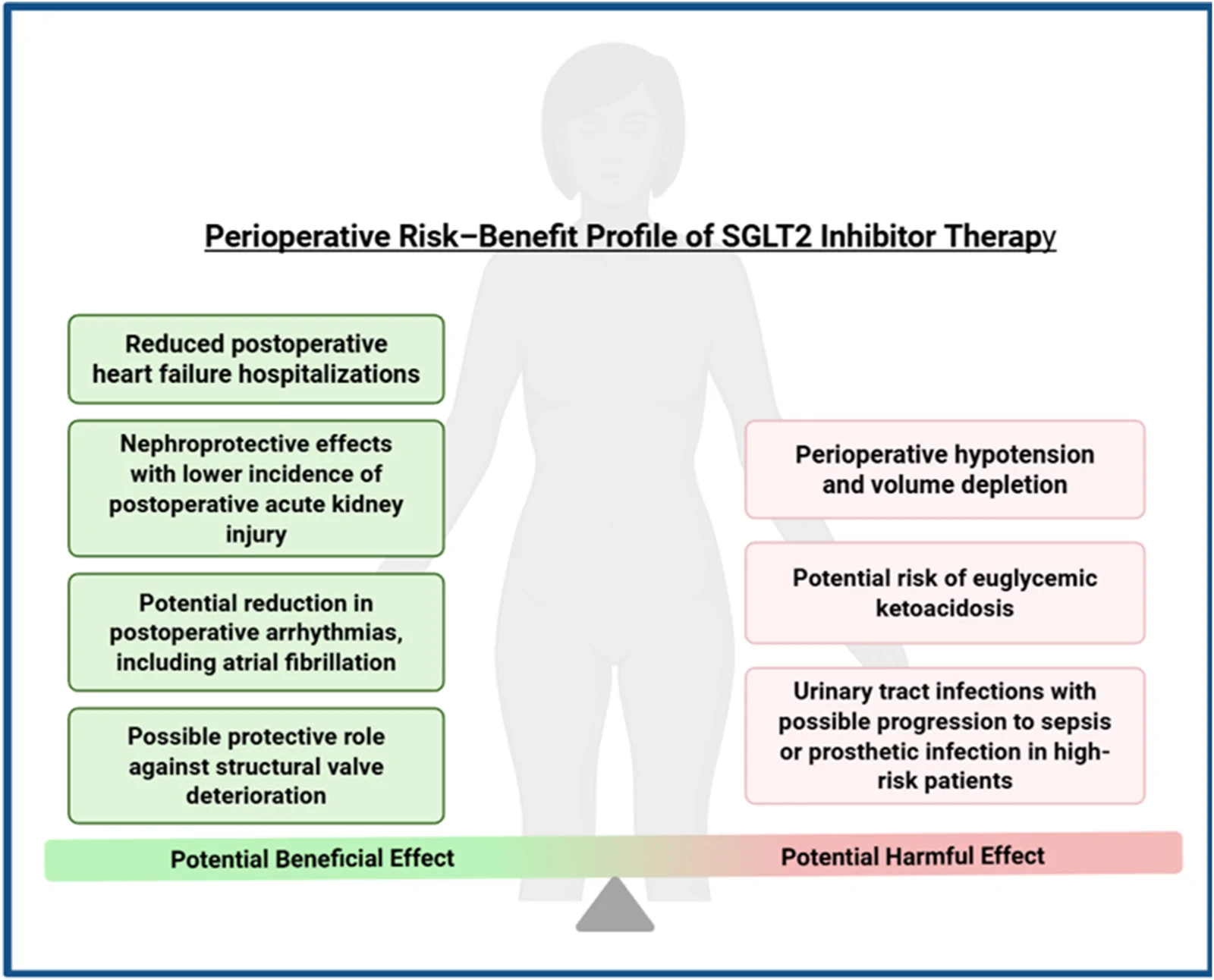
**Perioperativerisk-benefitprofile of SGLT2 inhibitors.** Summary of potential perioperative effects of SGLT2 inhibitor therapy in patients undergoing transcatheter and surgical valve interventions. Proposed benefits include reduced heart failure hospitalization, nephroprotection, lower arrhythmia risk, and possible protection against structural valve deterioration, whereas potential risks include hypotension and volume depletion, euglycemic ketoacidosis, and genitourinary infections in high-risk individuals.

Importantly, clinicians must remain vigilant regarding potential adverse effects, including volume depletion, urinary tract infection, and euglycemic ketoacidosis.^106^, ^107^, ^108^ In current practice, many perioperative protocols recommend withholding SGLT2 inhibitors approximately 3 to 4 days before surgery to mitigate ketoacidosis risk, with reinitiation considered once hemodynamic stability, renal function, and oral intake are restored.^109^ Further evidence is required to balance safety considerations with the potential cardiometabolic and renal benefits of therapy in patients undergoing surgical valve repair or replacement (Figure 3).

## Practical considerations for the interventional cardiologist

Effective integration of SGLT2 inhibitors into structural heart practice requires familiarity across the multidisciplinary Heart Team, including structural and interventional cardiologists, cardiac surgeons, HF specialists, anesthesiologists, and perioperative care teams. As these agents become increasingly relevant in patients with VHD and structural interventions, clinicians should understand their guideline-based indications, expected benefits, periprocedural management, contraindications, and potential adverse effects. Standardized institutional protocols may facilitate appropriate patient selection, avoid unnecessary perioperative discontinuation or delayed reinitiation, and ensure coordinated monitoring of renal function, volume status, blood pressure, and metabolic complications.

Given the limited and heterogeneous nature of the available evidence, SGLT2 inhibitors should not currently be initiated solely for VHD or with the expectation of modifying valvular disease progression. Rather, these agents should be prescribed according to established guideline-directed indications, particularly in patients with HF, chronic kidney disease, or diabetes mellitus, including those with concomitant VHD. Patients with elevated natriuretic peptide levels, recurrent HF hospitalizations, or residual myocardial dysfunction may represent particularly high-yield populations for therapy. Conversely, caution should be exercised in patients with severe hypotension, acute kidney injury, recurrent genitourinary infections, advanced frailty with limited oral intake, or a history of ketoacidosis.

In patients anticipated to undergo transcatheter or surgical valve intervention, optimization of guideline-directed medical therapy, including SGLT2 inhibitors when clinically indicated, should be considered as part of a comprehensive preprocedural assessment. This is particularly relevant in patients with secondary MR being evaluated for the M-TEER procedure, as part of HF optimization before intervention. However, currently available evidence does not support delaying an otherwise indicated intervention solely to assess response to SGLT2 inhibitor therapy. Following valve intervention, SGLT2 inhibitors may be initiated or resumed once the patient is hemodynamically stable, euvolemic, tolerating oral intake, and free of acute kidney injury or other contraindications. While these agents may represent a valuable adjunct to contemporary structural heart care, treatment decisions should remain individualized and incorporated within a multidisciplinary Heart Team approach.

## Real-world considerations and barriers

Despite favorable clinical outcomes, several practical barriers may limit the widespread adoption of SGLT2 inhibitors in structural heart populations. Medication cost and insurance coverage remain important considerations, particularly among elderly patients with multiple comorbidities and high medication burden. Adherence may also be challenging due to polypharmacy, cognitive impairment, or limited healthcare access.

Clinicians should routinely monitor volume status, renal function, and blood pressure following initiation, particularly in elderly patients receiving concomitant diuretic therapy. Patient education regarding genital and urinary tract infections, volume depletion, and recognition of symptoms suggestive of ketoacidosis is essential. These considerations highlight the importance of individualized risk-benefit assessment when incorporating SGLT2 inhibitors into comprehensive structural heart disease management.

## Limitations of current evidence

The current evidence supporting SGLT2 inhibitor therapy in VHD and structural heart interventions remains heterogeneous and should be interpreted cautiously. The strongest data are available in the post-TAVR population, where randomized evidence supports a reduction in worsening HF events among selected high-risk patients, although follow-up remains limited and long-term effects on survival, remodeling, and valve durability are uncertain. Evidence in functional MR is moderate and primarily supported by small randomized trials demonstrating improvements in MR severity and remodeling parameters, but these studies remain limited by modest sample sizes, imaging-focused endpoints, and limited follow-up. In contrast, evidence for degenerative AS progression, bioprosthetic valve degeneration, TR, rheumatic MS, and post–M-TEER outcomes is lower quality and largely derived from observational analyses or small early-phase studies, with important limitations including treatment-selection bias, residual confounding, immortal time bias, coding-based exposure definitions, and endpoint heterogeneity.

## Future directions

Despite emerging mechanistic insights and early clinical signals, the role of SGLT2 inhibition in VHD remains incompletely defined. There is a substantial unmet need for high-quality observational studies and adequately powered randomized clinical trials across the spectrum of VHD.

Medical therapies capable of modifying disease progression in moderate valvular disease—particularly aortic stenosis—remain limited. Randomized trials evaluating SGLT2 inhibitors in this population may help clarify whether these agents can influence disease progression and associated clinical outcomes. Similarly, dedicated studies in patients with TR are warranted, given the growing recognition of the benefits of guideline-directed medical therapy in this population.

Future investigations should also evaluate the role of SGLT2 inhibition in patients undergoing transcatheter valve interventions, including mitral and tricuspid transcatheter edge-to-edge repair (M-TEER and T-TEER), particularly in light of emerging supportive data from other structural heart intervention populations.^95^ In parallel, further evidence is needed to define the safety, efficacy, optimal timing, and patient selection for SGLT2 inhibitor therapy in individuals undergoing surgical valve interventions.

Collectively, such data will be essential to inform multidisciplinary Heart Team decision-making and guide the integration of SGLT2 inhibition into the broader medical and procedural management of patients with VHD.

## Conclusion

SGLT2 inhibitors have emerged as an important therapeutic class with potential relevance across the spectrum of VHD. Beyond their established systemic hemodynamic and metabolic effects, experimental and translational data suggest that SGLT2 inhibitors may modulate cellular pathways involved in inflammation, oxidative stress, myocardial fibrosis, and adverse remodeling. Early clinical studies and emerging randomized data also suggest potential benefit in selected VHD populations, including patients undergoing transcatheter valve interventions. However, much of the VHD-specific evidence remains derived from observational analyses, small clinical studies, and mechanistic investigations. Therefore, conclusions regarding valve-specific disease modification, valve durability, and periinterventional outcomes should be considered hypothesis generating and require confirmation in adequately powered randomized clinical trials. As mechanistic insights increasingly align with emerging clinical signals, SGLT2 inhibitors may represent a promising adjunctive therapy in the multidisciplinary management of structural heart disease, with future investigations needed to define optimal patient selection, timing of initiation, and integration with structural interventions.

## CRediT authorship contribution statement

**Haytham Allaham:** Writing – review & editing, Writing – original draft, Visualization, Data curation, Conceptualization. **C. Michael Gibson:** Supervision. **Aloke Finn:** Writing – review & editing, Supervision. **Imad Alhaddad:** Supervision. **Anuj Gupta:** Writing – review & editing, Supervision.
